# Ultrasonography-triggered diagnosis of putrid, ulcero-phlegmonous, hemorrhagic appendicitis and periappendicitis with an atypical symptom pattern: a case report

**DOI:** 10.1186/s40779-016-0088-z

**Published:** 2016-06-27

**Authors:** Hagen Frickmann, Sven A. Jungblut

**Affiliations:** Department of Tropical Medicine at the Bernhard Nocht Institute, German Armed Forces Hospital of Hamburg, Hamburg, Germany; Institute for Microbiology, Virology and Hygiene, University Medicine Rostock, Rostock, Germany; Practice Dr. Jungblut, Frankfurt/Main, Germany

**Keywords:** Acute appendicitis, Ultrasound, Oligosymptomatic, Surgery

## Abstract

**Background:**

Asymptomatic and oligosymptomatic appendicitis are rare and challenging diagnoses that should not be missed.

**Case presentation:**

A young female patient presented with mild to moderate pain in the middle and lower abdomen, and the results of physical examination, including digital rectal examination, were otherwise non-contributory. Ultrasonography demonstrated a marked increase of the outer appendiceal diameter up to 12 mm and a trace of free liquid around the terminal ileum. Subsequent surgical exploration and histological examination allowed for a final diagnosis of putrid, ulcero-phlegmonous, hemorrhagic appendicitis and periappendicitis.

**Conclusions:**

Ultrasonography is increasingly used for the diagnosis of appendiceal inflammation, particularly in military medical settings. Increases in the outer appendiceal diameter up to >6 mm under compression have recently been demonstrated to be indicative of acute appendicitis. At a minimum, in cases with doubtful physical examination results, ultrasonography should be considered as an element in the diagnosis of acute appendicitis.

## Background

The management of appendicitis is a standard situation for military surgeons. Among active component members of the U.S. Armed Forces, the overall incidence of appendicitis was 18.4 per 10,000 person-years between 2002 and 2011 [1]. Acute appendicitis is also among the frequent causes of surgical interventions during deployment [2, 3]. The high perforation rates abroad [2] typically result from incorrect initial diagnoses or treatments. During the missions of the U.S. Armed Forces in Iraq and Afghanistan, appendicitis was among the most frequent causes of medical evacuations [1].

The use of ultrasound scanning of the abdomen for the diagnosis of appendicitis has previously been evaluated. In cases of acute appendicitis, the outer appendiceal diameter increases to 7.9 ± 2.0 mm compared with a typical outer appendiceal diameter value of 4.5 ± 1.2 mm for healthy populations. Further, periappendiceal inflamed fat is frequently detected by ultrasound scanning in patients with appendicitis [4].

Here, we describe a rare case of progressed appendicitis that nevertheless presented with mild to moderate symptoms and was diagnosed based only on ultrasound scanning results.

## Case presentation

### Clinical history

A 21-year old female patient presented at the emergency department of a hospital with progressive pain in the middle and lower right abdomen, nausea and vomiting. The symptoms began 12 h prior to her arrival. Defecation and stool consistency were non-contributory. Fever and chills were denied. Lactose intolerance was the only reported pre-existing condition.

### Physical examination

The rectal temperature of the patient was 37.4 °C. The patient was alert and oriented. The described pain in the middle and lower right abdomen could be induced by pressure. Apart from these findings, the physical examination was non-contributory.

Specifically, the patient’s abdomen was soft, and there were no signs of guarding or peritoneal signs. Auscultation of the abdomen was non-contributory. A digital rectal examination did not provoke any pain in the recto-uterine pouch. The sphincter tone was normal. The stool was brown and did not exhibit any signs of blood or viscous secretions. The reported pain was noticeably decreased following novaminsulfon infusion. Overall, the patient appeared to be only marginally compromised, and dismissal from the emergency department was therefore considered.

### Routine laboratory results

A marked increase in the white blood cell count to 17.8 × 10^9^/L (ref.: 3.8 × 10^9^/L – 9.8 × 10^9^/L) was observed, whereas the C-reactive protein level was non-contributory at 2.4 mg/L (ref.: <5). A slightly increased free tetraiodothyronine level of 15.7 pmol/L (ref.: 7.7 – 14.2) was observed as an incidental finding. All other assessed laboratory parameters were normal.

### Ultrasonography of the abdomen

**Fig. 1:**
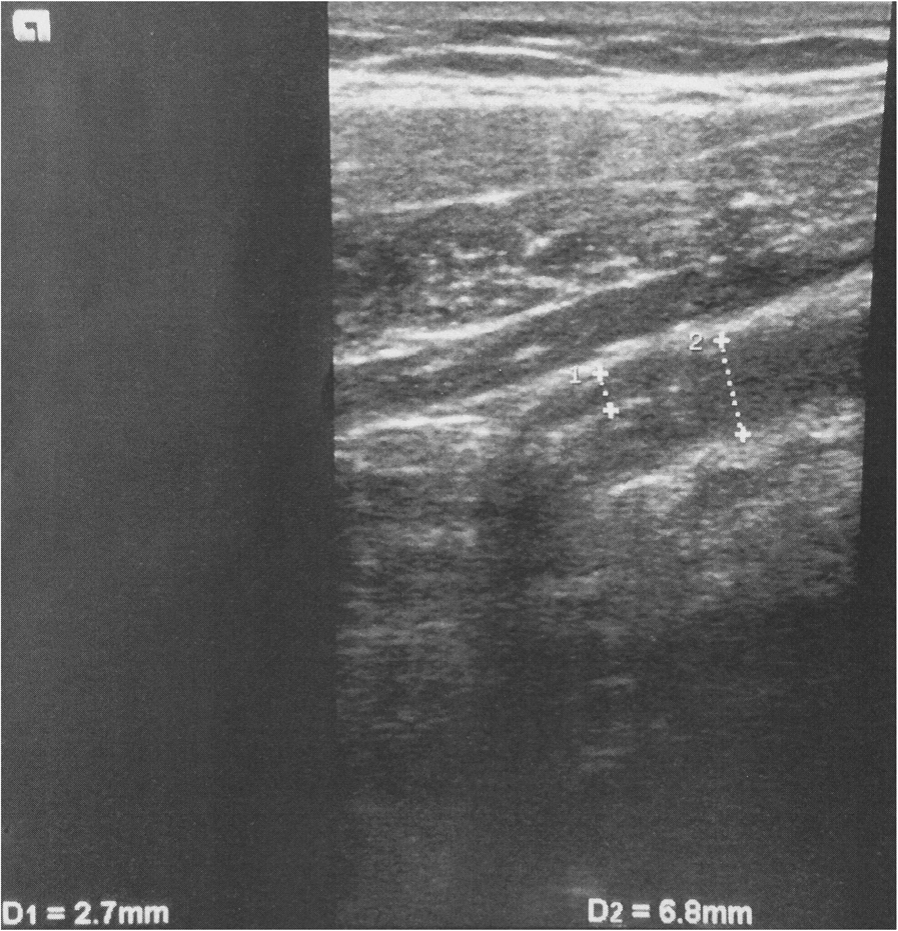
Ultrasonography of the abdomen. An ultrasound-triggered suspicion of acute appendicitis was based on the free liquid around the terminal ileum, the intestinal cockade sign and the increase of the wall thickness of the proximal appendix to 3 mm. D1 (2.7 mm) ist equal to the increased wall thickness with weak echo signal intensity. D2 (6.8 mm) is equal to the diameter of the inflamed appendix in its proximal part

There was a trace of free fluid around the terminal ileum. Further, an intestinal cockade sign was visible, and the size of the appendix wall was increased to 3 mm (Fig. 1). In the distal parts of the appendix, the outer appendiceal diameter was increased up to 12 mm.

The overall accuracy of ultrasonography for the identification of acute appendicitis was recently estimated at approximately 90 % based on an analysis of a small cohort of 60 patients [5]. In this study, an appendiceal diameter >6 mm under compression was considered to be the cut-off point for a reliable diagnosis [5]. On French submarines, ultrasonography of the abdomen is routinely performed in cases of suspected appendiceal abscesses, and the accuracy is high [6].

### Diagnosis and therapy

**Fig. 2:**
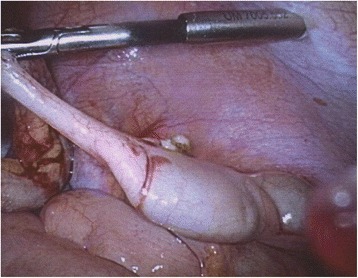
Intraoperative situs with an inflamed phlegmonous appendix. The suboptimal shooting angle and range make the observation of the anatomical relations difficult, which reflects the real-life surgical situation

Based on the ultrasound results, a diagnosis of acute appendicitis was made. Laparoscopic surgical intervention allowed for the extirpation of an inflamed, phlegmonous appendix (Fig. 2).

The operation and postoperative management were without any complications.

### Histological assessment

Putrid, ulcero-phlegmonous, hemorrhagic appendicitis and peri-appendicitis with putrid fibrinous serositis and fecal impaction were diagnosed. A partial spread of the inflammatory process beyond the surgical margins was observed.

## Conclusions

Skilled use of abdominal ultrasonography can serve critical diagnostic purposes for forward deployed troops when other modalities, such as computed tomography, are not available due to the austere settings. In such situations, abdominal ultrasonography is particularly important for young females for whom the differential diagnoses of abdominal and pelvic pain are much broader [7, 8] and including gynecological diagnoses, such as pelvic inflammatory disease [9, 10], torsion, ectopic pregnancy [11], cystitis [12], other infections [13], and pelvic pain of unclear etiology [12].

The peculiarity of the present case is that ultrasonography provided the most important information for the diagnosis of appendicitis. Oligosymptomatic and asymptomatic cases of appendicitis are both rare [14] and challenging in terms of differential diagnosis. In the present case, mild to moderate symptoms without pain in the recto-uterine pouch were revealed by the physical examination despite an advanced inflammatory process that affected the patient’s mesentery. Without the ultrasonography results, the patient, who presented with mild to moderate appendicitis, would have been sent home, and a watch-and-wait approach would have been employed. This approach might have resulted in perforation and the associated complications. However, ultrasonography demonstrated an increased appendiceal diameter that far exceeded the >6 mm cut-off point that has recently been suggested [5]. Furthermore, a trace of free fluid around the terminal ileum was observed, which supported the diagnosis. Although the importance of ultrasound scanning for the diagnosis of appendicitis is well established, the fact that a severely progressed state of inflammation was associated with symptoms that were sufficiently mild that the patient would have been sent home without the ultrasound result is quite unusual and should be kept in mind.

Both computed tomography (CT) and ultrasound scanning (US) have been demonstrated to be useful modalities in the diagnosis of acute appendicitis. Both techniques are used to reduce the number of unnecessary surgical interventions [15], but US also has the advantage of providing results without exposure to ionizing radiation [15]. The use of US scoring systems can contribute to a reduction in the use of CT scanning while ensuring the diagnosis [16]. Nonvisualization of the appendix on US is a highly predictive sign of the absence of appendicitis with an accuracy of 94.3 % [17], particularly in children when leukocytosis is absent [18]. The secondary signs of hyperemia, i.e., fluid collection and the presence of an appendicolith, exhibit 96 % specificity and 88 % accuracy for the presence of appendicitis in cases of otherwise equivocal ultrasound results [19]. Next to the visualizations of the appendix and periappendiceal fluid, an appendiceal diameter >6 mm, an appendix wall thickness >2 mm, and indirect associated signs, such as an increased white blood cell count and an increased polymorphonuclear percentage, are considered to be predictors of appendicitis [20]. However, perforations in cases of acute appendicitis are likely to go undetected based on US [21].

CT is the most accurate imaging modality for cases of suspected appendicitis and should be considered in cases of questionable US findings when a CT scanner is available [22]. If there are contraindications for the use of contrast enhancers, noncontrast CT also exhibits a high diagnostic accuracy for the detection of appendicitis [23]. The interpretation of scores based on a specific cutoff points, such as the Alvarado score, ease the standardized interpretation of CT results [22]. However, the ionizing radiation associated with CT is one of its disadvantages. Furthermore, even when CT is employed, the detection of perforation signs is not easy in the early stages of the process [24], particularly when an abscess and phlegmon are still absent. Potential perforation should be considered if extraluminal air bubbles, increased wall thickness, or the presence of an intraluminal fecalith are observed on CT imaging [25]. In cases of borderline appendix sizes, the combined detection of wall thickening and the absence of intraluminal air on CT imaging has been confirmed to be a reliable predictor of appendicitis [26]. Compared with US, CT-measured appendiceal diameters may vary by 1–2 mm, and this variation needs to be considered during the interpretation of the results [27]. Notably, the use of CT has not been demonstrated to be associated with better outcomes in patients with appendicitis [28, 29].

If ionizing radiation is to be avoided, magnetic resonance imaging involving gadolinium-enhanced and T_2_-weighted images has been demonstrated to be an expensive but useful alternative for the detection of acute appendicitis [30, 31]. Recently, the measurement of the elastic modulus values via shear wave elastography has been suggested as a new diagnostic approach for distinguishing between inflamed and normal appendices [32]. Larger studies would likely be useful to assess the clinical effects of this procedure.

In cases of less advanced oligosymptomatic appendicitis, colonscopy is an alternative procedure that might support the diagnosis [33]. In contrast, the disease-associated results of laboratory diagnostic procedures may be non-specific. Cyclic neutropenia has been described in a previous case of asymptomatic appendicitis [34]. In the presently described case, leukocytosis suggested the presence of an inflammatory process.

As demonstrated by a case of bilateral ureteral obstruction following asymptomatic appendicitis [35], the consequences of the delayed diagnoses of oligosymptomatic and asymptomatic appendicitis can be severe. Experience with ultrasonography of the abdomen may contribute to the prevention of similar unfavorable clinical courses.

## Consent

Written informed consent for the publication of this case report and any accompanying images was obtained from the patient. A copy of the written consent is available for review by the Editor-in-Chief of this journal.

## Abbreviations

CT, computed tomography; US, ultrasound scanning
